# Advances in Hodgkin Lymphoma: Including the Patient’s Voice

**DOI:** 10.3389/fonc.2022.855725

**Published:** 2022-02-25

**Authors:** Christine Moore Smith, Debra L. Friedman

**Affiliations:** ^1^ Division of Pediatric Hematology/Oncology, Vanderbilt University Medical Center, Nashville, TN, United States; ^2^ Vanderbilt-Ingram Cancer Center, Vanderbilt University Medical Center, Nashville, TN, United States

**Keywords:** Hodgkin, lymphoma, patient-reported outcomes, immunotherapy, survivorship, targeted therapy, Hodgkin lymphoma (HL)

## Abstract

Since the initial treatment with radiation therapy in the 1950s, the treatment of Hodgkin lymphoma has continued to evolve, balancing cure and toxicity. This approach has resulted in low rates of relapse and death and fewer short and late toxicities from the treatments used in pursuit of cure. To achieve this balance, the field has continued to progress into an exciting era where the advent of more targeted therapies such as brentuximab vedotin, immunotherapies such as PD-1 inhibitors, and chimeric antigen receptor T-cells (CAR-T) targeted at CD30 are changing the landscape. As in the past, cooperative group and international collaborations are key to continuing to drive the science forward. Increased focus on patient-reported outcomes can further contribute to the goal of improved outcomes by examining the impact on the individual patient in the acute phase of therapy and on long-term implications for survivors. The goals of this review are to summarize recent and current clinical trials including reduction or elimination of radiation, immunotherapies and biologically-targeted agents, and discuss the use of patient-reported outcomes to help discern directions for new therapeutic regimens and more individualized evaluation of the balance of cure and toxicity.

## Introduction

Hodgkin Lymphoma (HL) is a malignant lymphoma with an impact spanning both the pediatric and adult populations. Cases occur in a bimodal distribution with peak in the adolescent and young adult (AYA) population, with varying definitions, but commonly considered to encompass the ages of 15-39 years (1–3). Within pediatrics, the incidence of HL is 12.2 per million for children under age 20, but 32 per million in ages 15-19, and highest between 20-24 years at 45 per million (4, 5).

With current treatment options, HL has a high cure rate. After the advent of successful treatment with radiotherapy (RT) and then subsequently chemotherapy, death rates from HL have declined since 1975, with an additional impressive decrease of 4% per year from 2008 to 2017. Recent data highlight excellent overall survival (OS) of 87% at 5 years across the age span and 95% for pediatric patients (1, 6). The failure rate of first-line therapies has similarly declined with 90% event free survival (EFS) in early stage disease and 80-85% EFS in advanced staged disease (7). Clinical trials have been essential in contributing to these improvements. With such high survival rates, focus over the last three decades has shifted to reducing both the acute and long-term effects of treatment while maintaining long-term EFS and OS. This focus is important as treatment of relapsed/refractory disease requires additional exposure to toxicity through salvage regimens, RT, and potentially high dose therapy with autologous hematopoietic cell transplant (HDT/AHCT) (6).

Varied multimodal approaches to achieve these goals have been studied in clinical trials, albeit without clear consensus on the best approach. As we move forward with efficacious regimens, we continue to learn how to best incorporate, prioritize, and sequence the use of newer agents. This can be enhanced, in part, by incorporating patient-reported outcomes (PROs). This is an exciting step toward understanding the patient-level impact of regimens on EFS, OS, and tolerability of acute and long-term effects of treatment.

## Risk Adapted Therapy in Frontline Clinical Trials

Clinical trials in the last two decades have explored effective multiagent chemotherapy regimens for response-based risk adaptation. For patients with early responses to chemotherapy, most regimens balance curative goals with late toxicity by omitting or reducing RT. Reduction in radiation doses and fields spare normal tissues and are anticipated to decrease radiation-associated adverse long-term health effects (8). The chemotherapy backbones decreased alkylators and anthracyclines to minimize long-term adverse effects of these agents including fertility issues, secondary malignant neoplasms, and cardiotoxicity. While these have been central goals, approaches have varied somewhat in different pediatric collaborative groups including the Children’s Oncology Group (COG), the St Jude-Stanford-Dana Farber Consortium, the German Paediatric Haematology-Oncology Group, and the European Network for Paediatric Hodgkin Lymphoma (EuroNet-PHL), among others. With the advent of the National Cancer Institute sponsored National Clinical Trials Network (NCTN), there is also the opportunity for further collaboration between pediatric and adult cooperative groups in the United States.

**Table 1** reviews some of these more recent studies from pediatric and adult cooperative groups (9–16). The next generation of investigation builds upon these studies with incorporation of more biologically-targeted approaches.

**Table 1:** Recent Cooperative Group Studies.

Cooperative Group/Study	Goals	Chemotherapy and	Outcomes and Notes
		Radiotherapy	
**COG** **AHOD0031** (9)	Response-based risk-adaptation for reduction of RT; evaluate intensification of chemotherapy for intermediate-risk patient	ABVE-PC +/- DECA	EFS = 85%; OS = 97.8% For early responders, IFRT did not significantly change EFS. Chemotherapy intensification to DECA versus no DECA did not significantly change EFS for slow responders (9).
		IFRT to 21 Gy based on disease at presentation if not in CR at early response assessment	
**COG** **AHOD0431** (10)	Response-based risk-adaptation for reduction of chemotherapy and RT for low-risk patients with an integrated chemotherapy plus RT salvage regimen	*Frontline*: AVPC *Relapse*: IV + DECA	If CR on FDG-PET scan (PET) after 1 cycle of chemotherapy, the 4-year EFS was 88.2% versus 68.5%. Patients with low stage mixed cellularity histology had an excellent EFS of 95.2%
		IFRT to 21 Gy based on disease at presentation if not in CR at early response assessment or at relapse	
**COG** **AHOD0831** (11)	Response-based risk-adaptation for reduction of cumulative alkylators and RT in high-risk patients	ABVE-PC +/- IV	5-year EFS (all patients) = 79.1%; Rapid early response EFS = 83.5%; Slow early response EFS = 73.2%. EFS was below the prespecified target for the trial.
		IFRT to 21 Gy to initial bulky disease and sites of slow response	
**EuroNet-PHL** **C1** (12)	Comparison of consolidation regimens and reduction of RT; results published for intermediate and high-risk groups	OEPA + COPP vs COPDAC	49% of intermediate and 35% of high-risk with adequate response to chemotherapy and did not have subsequent RT with 5-year EFS = 90.1%. Patients on the COPP arm had EFS of 89.9% and COPDAC 86.1%.
		RT to 19.8 Gy at all initially involved tumor sites for patients with inadequate response to chemotherapy alone; additional 10 Gy boost to bulky sites or slow response	
**EuroNet-PHL** **C2** (13)	Evaluate intensification of chemotherapy from COPDAC-28 to DECOPDAC-21 and reduce use of RT by targeting FDG-avid sites of disease at end of chemotherapy	OEPA +/- COPDAC-28 vs DECOPDAC-21 in certain cases	Results not yet available. Notably moved toward the more modern definition of Deauville positivity of 4 and 5, which will increase the number of patients eligible for elimination of RT.
		Randomization depending on risk group and early and late response assessments; dose ranges from 19.8 Gy to 30 Gy	
**ECOG** **E2496** (14)	Compared chemotherapy regimens for superiority of Stanford V over ABVD	ABVD vs Stanford V	No significant difference in response rate or in failure-free survival. Toxicity was reported to be similar between the two arms. The authors concluded that ABVD should remain the standard of care.
		RT to 36 Gy for all bulky mediastinal adenopathy; RT on Stanford V arm to 36 Gy for lesions > 5 cm or macroscopic splenic disease	
**SWOG** **S0816** (15, 16)	Evaluate intensification of therapy if PET2 positive	ABVD +/- eBEACOPP	PET2 was negative for 82% of patients; 5-year PFS = 76% for PET2 negative versus 66% for PET2 positive.
		None	

Recent collaborative group clinical trials with response-based risk-adjusted chemotherapy and radiotherapy (RT). ABVE-PC: doxorubicin, bleomycin, vincristine, etoposide, prednisone, cyclophosphamide; DECA: dexamethasone, etoposide, cisplatin, cytarabine; IV: vinorelbine, ifosfamide; AVPC: doxorubicin, vincristine, prednisone, cyclophosphamide; OEPA: vincristine, etoposide, prednisone, doxorubicin; COPP: cyclophosphamide, vincristine, prednisone, procarbazine; COPDAC(-28): cyclophosphamide, vincristine, prednisone, dacarbazine; DECOPDAC-21: 21 day cycle of COPDAC; ABVD: doxorubicin, bleomycin, vinblastine, dacarbazine; Stanford V: doxorubicin, vinblastine, chlormethine, vincristine, bleomycin, etoposide, prednisone; IFRT: involved field radiotherapy.

## Advances in Treatment With Brentuximab Vedotin

Brentuximab vedotin (Bv) is an antibody-drug conjugate that targets delivery of monomethyl auristain E to cells expressing CD30 such as the Reed Sternberg cells in classical HL.

Initially studied in the relapsed/refractory setting in ages ≥12 including adults, a study of Bv monotherapy showed efficacy with CR in 38% and some durable remissions while overall being well-tolerated (17, 18).

Pediatric and AYA regimens have combined Bv with traditional cytotoxic chemotherapy in the relapsed/refractory disease setting. For patients ≤30, COG AHOD1221 evaluated Bv with gemcitabine and reported 67% of patients achieved CR after 4 cycles when including patients meeting modern Deauville score criteria. *Ad hoc* analysis showed 1-year OS of 95% (19). Bv has also been studied with bendamustine in several trials including the pediatric and AYA populations with CR rates of 66-79% and 2 and 3-year progression free survival (PFS) of 62.6-69.8% (20–23).

In pediatrics, several studies have also incorporated Bv in frontline treatment. A single-arm trial led by the St. Jude-Stanford-Dana-Farber Consortium for ages ≤18 evaluated the safety and efficacy of Bv for high-risk patients in a backbone of A-EPA/CAPDac (Bv, etoposide, prednisone, doxorubicin, cyclophosphamide, dacarbazine). Results included 3-year EFS of 97.4%, and 35% of patients were early responders avoiding need for RT. The study highlights tolerability of Bv and effectiveness of residual node radiation (24). AHOD1331, a trial by the COG for ages 2-21, completed accrual of high-risk patients treated with a backbone of ABVE-PC (doxorubicine, bleomycin, vincristine, prednisone, cyclophosphamide) compared with Bv substitution for bleomycin. Need for involved site RT was determined by PET response. Data release and analyses are expected in the near future (25).

For frontline trials in adult patients, the ECHELON-1 trial compared the standard of ABVD (doxorubicin, bleomycin, vinblastine, dacarbazine) with A-AVD (Bv, doxorubicin, vinblastine, dacarbazine) for advanced stage disease in patients ages ≥18. The 3-year PFS was superior for A-AVD versus ABVD (83.1% versus 76%) (26, 27).

The most notable dose limiting toxicity of Bv is neuropathy which is reported subjectively by patients and has been shown to be tolerable and reversible in most trials. Reliance on the patient experience for toxicity reporting exemplifies how standardized PROs can help measure tolerability to determine which regimens best balance efficacy and toxicity (24, 27, 28).

## Advances in Treatment With Immune Checkpoint Inhibitors

HL cells have overexpression of programmed death-1 (PD-1) ligands 1 and 2 due to alterations in the 9p24.1 locus, and PD-L1 is also expressed in tumor associated macrophages making immune checkpoint inhibitors (ICIs), specifically anti-PD-1 monoclonal antibodies, promising agents for investigation in HL (29–31). Cytotoxic T lymphocyte-associated protein 4 (CTLA-4) blockade is an alternate approach which activates peritumoral T cells to overcome T cell exhaustion in the tumor microenvironment. Ipilimumab is a monoclonal antibody targeting CTLA-4 currently being evaluated both alone and in combination as it has shown synergy with nivolumab in other cancers such as melanoma (32, 33). A number of studies first conducted among adults have demonstrated promising results in HL, and pediatric trials are now underway to ascertain if similar results can be attained in younger patients.

CheckMate 205 evaluated nivolumab, a PD-1 inhibitor, in several cohorts of patients ages ≥18, including relapsed/refractory disease as well as upfront with nivolumab monotherapy followed by a combination regimen of N-AVD (nivolumab, doxorubicin, vinblastine, dacarbazine). There were good overall response rates (ORR) of 71% in the relapsed/refractory cohort and 21% CR. Patients in CR had a longer median PFS (37 months) versus partial response (15 months). Upfront responses were higher with 67% achieving CR and, at time of the report, 92% PFS at a median of 9 months (31, 34–36).

KEYNOTE-087 evaluated pembrolizumab, a PD-1 inhibitor, in patients ages ≥18 with relapsed/refractory disease in multiple cohorts, with good ORR of 71.9% though only 27.6% achieved CR with a median duration of response of 16.5 months (37).

To evaluate combinations of ICIs, E4412, led by the ECOG-ACRIN Cancer Research Group, evaluated combinations of Bv with nivolumab, Bv with ipilimumab, or triplet therapy in ages ≥18 and has now expanded recruitment through the COG to include children and adolescents ≥12 with relapsed/refractory disease (38). The ipilimumab group showed 76% ORR, nivolumab 89%, and triplet therapy 82%; the PFS and OS are not yet fully reported. The triplet therapy had more adverse events than two agent combinations in the adult population, but this will remain to be seen in pediatrics and could inform which regimens are best to pursue in future trials (39, 40).

In pediatrics, the COG evaluated nivolumab as a single agent in relapsed or refractory solid tumors and lymphomas in ADVL1412 which showed 3 of 10 patients with HL, all of whom had PD-L1 expression, had responses (41).

Frontline collaborative studies now exist between pediatric and adult study groups (COG, EuroNET-PHL, NCTN, SWOG Cancer Research Network, Alliance for Clinical Trials in Oncology, ECOG-ACRIN, and NRG Oncology) to evaluate PD-1 inhibitors with chemotherapy in combined adult-pediatric populations. While open for all stages for adult patients, pediatric patients ages 3-25 can enroll on the low-risk arm of KEYNOTE-667, also known in COG as AHOD1822, evaluating the addition of pembrolizumab for patients with less than CR after 2 cycles of ABVD (42). Led by SWOG, S1826 randomizes patients ages ≥12 with higher risk disease between A-AVD versus N-AVD (43).

As these agents are now being more widely used in both upfront and relapsed/refractory regimens, there is evidence that rechallenge with targeted agents such as Bv or ICIs can be efficacious and tolerable even if there has been progressive disease or prior dose-limiting toxicity related to the agent (39, 44, 45).

## Additional Advances in Treatment of Relapsed and Refractory Disease

Refractory HL occurs in up to 5-10% of cases and 10-30% of patients will experience relapse, though these numbers are lower in pediatric only trials (39). Some of the factors affecting risk stratification include time to relapse, primary refractory disease, heavy pretreatment with radiation and/or chemotherapy, extranodal disease, higher stage/risk group, anemia, and B symptoms at relapse (39). Historically, combination chemotherapy regimens were the salvage approach for relapsed and refractory disease. More information about these chemotherapy regimens can be found in reviews by Voorhees and Beaven and in the National Comprehensive Cancer Network (NCCN) Clinical Practice Guidelines for Hodgkin lymphoma and Pediatric Hodgkin lymphoma (39, 46, 47). Disease that has previously been chemotherapy responsive is a positive prognostic factor for success of such regimens for recurrent disease. However, for those with chemotherapy refractory disease, salvage regimens utilizing chemotherapy alone are likely less effective. Thus, biologically-targeted agents such Bv, ICIs, chimeric antigen receptor T-cells (CAR T), and molecular targets are exciting options for those with recurrent or refractory disease (39).

Traditionally, HDT/AHCT has been considered the standard of care for most relapsed/refractory HL. However, this approach may be challenged somewhat as new biologically-targeted agents are incorporated. The EuroNet-PHL published recommendations regarding who may benefit from HDT/AHCT versus chemotherapy/immunotherapy and/or RT alone. They propose a risk stratification based on time to relapse (primary refractory/progression, early relapse 3-12 months, or late relapse after 12 months), significant prior treatment, stage at relapse, and response to salvage therapy (7). Complete metabolic response (CMR) is also a key component for prognosis with HDT/AHCT, though this can be complicated by the use of PD-1 inhibitors that can cause FDG-avidity leading to difficulty interpreting response on PET (44). A review by Harker-Murray highlighted similar risk factors to determine the utility of HDT/AHCT (48).

A phase II study for ages ≥18 evaluated a combination of pembrolizumab with gemcitabine, vinorelbine, and liposomal doxorubicin. The regimen was efficacious in achieving CR in 95% with few toxicities allowing continuation to HDT/AHCT and maintaining remission at a median of 13.5 months (49).

In studies with combined pediatric and adult patients, targeted agents are being combined with chemotherapy. The COG study AHOD1721 evaluated Bv with nivolumab for ages 5-30. This regimen was well-tolerated with 59% in CMR after 4 cycles. For those not in CMR, 2 cycles of Bv and bendamustine were added leading to 88% of patients achieving CMR prior to consolidative therapy with HDT/AHCT off study (50, 51).

Continued maintenance therapies with or without HDT/AHCT with ICIs and Bv are another strategy with encouraging efficacy data (28, 39, 52, 53). In adults, the AETHERA trial evaluated the use of Bv in patients ages ≥18 as maintenance therapy following HDT/AHCT and demonstrated improvement in PFS (52, 53). A trial of pembrolizumab post-HDT/AHCT in patients ages 20-69 showed 82% PFS at 2 years (54). In pediatrics, Bv has also been used after HDT/AHCT in ages 16-22. Retrospective analysis showed tolerability of Bv and 100% CR in 5 patients (28).

CAR T products directed at CD30 are being evaluated for safety and efficacy with early results showing variable responses in relapsed/refractory CD30 positive lymphomas. Various co-stimulatory domains are being evaluated to improve outcomes including CD28 and CD137. Different lymphodepletion regimens affect efficacy with fludarabine leading to the best outcomes (29, 32, 45, 55, 56). A study of CD30 CAR T cells showed an encouraging ORR of 72% and CR of 59% in heavily pretreated patients ages 17-69 (57). Alternatively, CAR T directed at CD123 is under investigation given expression in 50-60% of Reed-Sternberg cells and the tumor microenvironment (29, 58, 59).

Other targeted therapies based on the biology and epidemiology of HL include JAK inhibitors, lenalidomide, everolimus, mocetinostat, panobinostat, and vorinostat (29, 58). Additionally, preclinical studies are showing the restoration of the typical B cell phenotype to retrieve CD19 expression allows for targeting by CD19 specific agents like blinatumomab or CD19 CAR T cells. Alternatively, CD20 retrieval combined with arsenic trioxide restores CD20 and allows for targeting with anti-CD20 monoclonal antibodies (29, 60).

## Importance of Patient-Reported Outcomes in Frontline Care and Survivorship Care

Given the excellent disease outcomes, minimizing acute and late effects of therapy can help determine the best regimens for individual patients. Incorporation of PROs as secondary and exploratory aims in the setting of clinical trials can help inform comparison across studies based on efficacy and patient experience (61). PROs are self-reported using validated questionnaires and can encompass physical, social, and emotional impacts of disease and treatment (62). Incorporation of PRO measurement into cancer care and creation of newer tools have helped drive the field forward (61, 63–66).

Retrospectively, Johannsdottir reported 63% of Norwegian childhood lymphoma survivors treated from 1970-2000 reported psychosocial adverse health outcomes and 97% reported at least one physical adverse health outcome using the Medical Outcome Study short form-36, CTCAE version 4.0, the Hospital Anxiety and Depression Scale, and the Fatigue Questionnaire. The majority of patients underwent combined chemotherapy and RT with a trend toward chemotherapy-only patients reporting better general health than patients undergoing combined modality or RT alone (67).

Berkman described the inclusion of PROs in phase 3 clinical trials in HL including the AYA population between 2007-2020 using the European Organization for Research and Treatment of Cancer Quality of Life Core Questionnaire (QLQ-C30). Four trials (17.4%) included PROs, but none have yet published the results (62). This is something to look forward to as studies and data mature, and, as suggested by Leblanc, we should expect PRO aims to be published with the primary results of any trial (61). The COG has endorsed and prioritized PRO inclusion in trials, demonstrating the willingness of cooperative groups to both collect and analyze the data (62).

KEYNOTE-087 reported on PROs including reports of health-related quality of life metrics as well as the response rate data showing greater than 70% of questionnaires were completed. For health-related quality of life scoring using the QLQ-C30 and the EuroQoL Five Dimensions Questionnaire, all three cohorts had similar baseline scores and improvement in both functional and symptom domains after initiation of treatment. Those with partial or complete responses had more improvement in their PRO measures than patients with stable or progressive disease (68).

There are many PRO options that can be incorporated into clinical trials, making comparisons more challenging. Standardization of PROs and comprehensive data collection can provide valuable data to assist in therapeutic decision-making for individual clinicians, individual patients, and cooperative groups planning future clinical trials. With both upfront reporting during clinical trials and follow-up in a survivorship setting, PROs can help provide a meaningful comparison of regimens regarding patient experience during and following therapy.

One example of a gap in care that could be narrowed with PROs is evaluation of psychosocial stressors. Distress in HL has been reported to exceed 30%, and recent work by Troy revealed distress levels were highest during active treatment, related to patient stress and experience of disease and therapy (65, 69). Worry and nervousness were also reported as acute psychosocial stressors (3). Addressing unmet needs as reported directly by the patient in a timely manner during therapy and in long-term follow-up can help alleviate some of the burden of the treatment experience, and these experiences may not be identified easily by other means. PROs provide standardized and validated ways to collect this data which is potentially actionable at the patient level but also by collaborative groups to better understand complications of treatment that may warrant further investigation.

The patient report of physical symptoms is also important with common symptoms being fatigue, nausea, and pain (3). Long-term health effects of HL therapy have traditionally included pulmonary fibrosis, pneumonitis, heart disease, thyroid dysfunction, chronic fatigue, neurocognitive effects, osteoporosis, and sexual dysfunction (70). Tracking of and response to patient report of these symptoms and experiences will continue to grow as a critical component of clinical trials and optimization of care. This is particularly important with regimens reducing cytotoxic chemotherapy and RT and incorporating newer agents.

With newer agents, toxicity monitoring is essential, particularly in the pediatric population. Short and long-term toxicity may differ between adult and pediatric patients. ICIs have different toxicities than traditional cytotoxic chemotherapies including autoimmune hepatitis, thyroid dysfunction, pneumonitis, colitis, rashes, fatigue, infusion reactions, pyrexia, and more rarely neurologic, renal, ocular, and pancreatic toxicity (71). Given this diverse set of toxicities, incorporation of PROs and structured follow-up of survivors can identify the prevalence and severity of adverse effects in the AYA and pediatric patient as these agents are increasingly being used.

## Discussion

Despite many advances, there remains lack of consensus regarding the best management of HL, and priorities differ for which strategies to evaluate next in clinical trials. Better understanding the patient experience and outcomes through collaboration, clinical trials, and the use of PROs could be an important step forward to achieve the best outcomes and therapy options.

Following patients over the last several decades allowed us to recognize the long-term health effects of curative therapeutic approaches, and subsequently long-term follow-up is essential to avoid trading one toxicity for another. Harmonization efforts for supportive care and long-term follow-up recommendations are underway and will be beneficial to provide therapy-specific and risk-adapted monitoring for toxicities and effects of therapy (2, 6, 70, 72–74). Ehrhardt makes a compelling argument to assess and consider the risk of late toxicities, converting this risk assessment into actionable data for the choice of upfront therapy (6).

Moving forward, considering toxicities in clinical decision-making, continuing to evaluate new treatments through collaborative clinical trials, and formalizing assessment of PROs can help achieve the goals to reduce toxicity and maintain high rates of cure. Continued collaborations can help standardize risk assessment, data collection, and toxicity reporting so trials can more easily be compared. This data will be invaluable when choosing a treatment for an individual patient.

## Data Availability Statement

The original contributions presented in the study are included in the article/supplementary material. Further inquiries can be directed to the corresponding author.

## Author Contributions

CS and DF conceptualized, drafted, reviewed, and approved the final manuscript.

## Conflict of Interest

The authors declare that the research was conducted in the absence of any commercial or financial relationships that could be construed as a potential conflict of interest.

## Publisher’s Note

All claims expressed in this article are solely those of the authors and do not necessarily represent those of their affiliated organizations, or those of the publisher, the editors and the reviewers. Any product that may be evaluated in this article, or claim that may be made by its manufacturer, is not guaranteed or endorsed by the publisher.
